# Supplementary material for: Post-Treatment of Tannic Acid for Thermally Stable PEDOT:PSS Film
Source: Polymers (Basel). 2022 Nov 14;14(22):4908. doi: 10.3390/polym14224908 (PMC9692676; doi:10.3390/polym14224908)
Supplement: Supplementary file 1 [file polymers-14-04908-s001.zip › polymers-2007074-supplementary.pdf]

Supporting Information

Post-treatment of Tannic Acid for Thermally Stable PEDOT:PSS Film

In-Seong Hwang [1], Ju-Yeong Lee [1], Jihyun Kim [1], Na-Young Pak [2], Jinhyun Kim [1,*] and Dae-Won Chung [1,*]

1 Department of Chemical and Materials Engineering, University of Suwon, Hwaseong 18323, Korea

2 EverChemTech Co., Ltd., 38, Cheongwonsandan 7-gil, Mado-myeon, Hwaseong 18543, Korea

* Correspondence: kim767@suwon.ac.kr (J.K.); dwchung@suwon.ac.kr (D.-W.C.); Tel.: +82-31-220-2352 (J.K.); Tel.: +82-31-220-2156 (D.-W.C.)

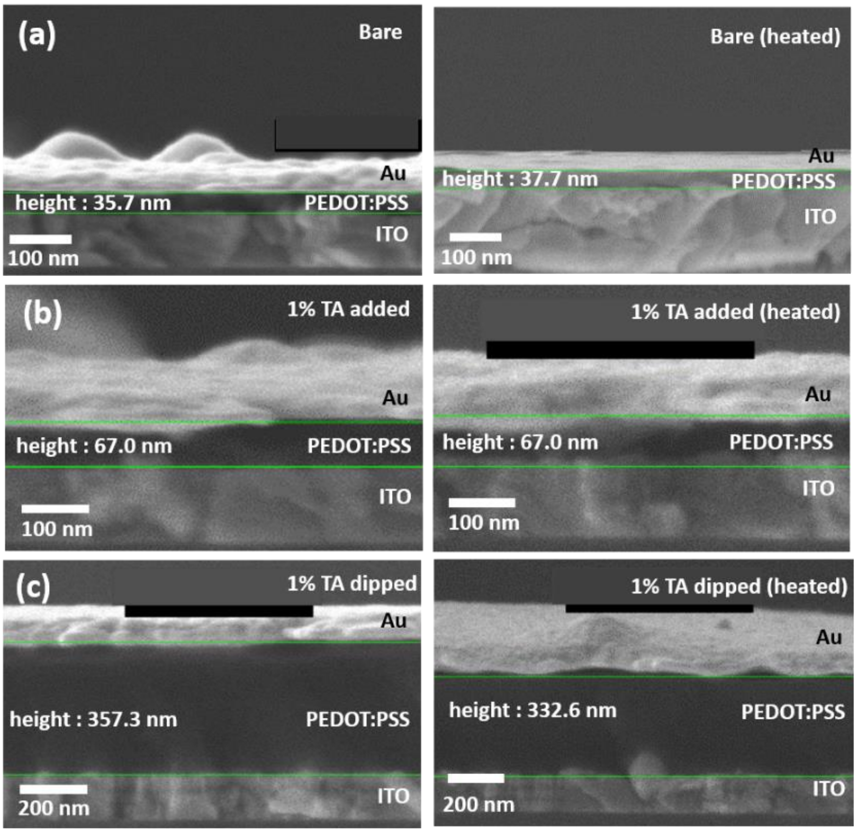

Figure S1. Cross-sectional SEM images of (a) PEDOT:PSS, (b) TA added PEDOT:PSS, (c) TA-dipped PEDOT:PSS films.

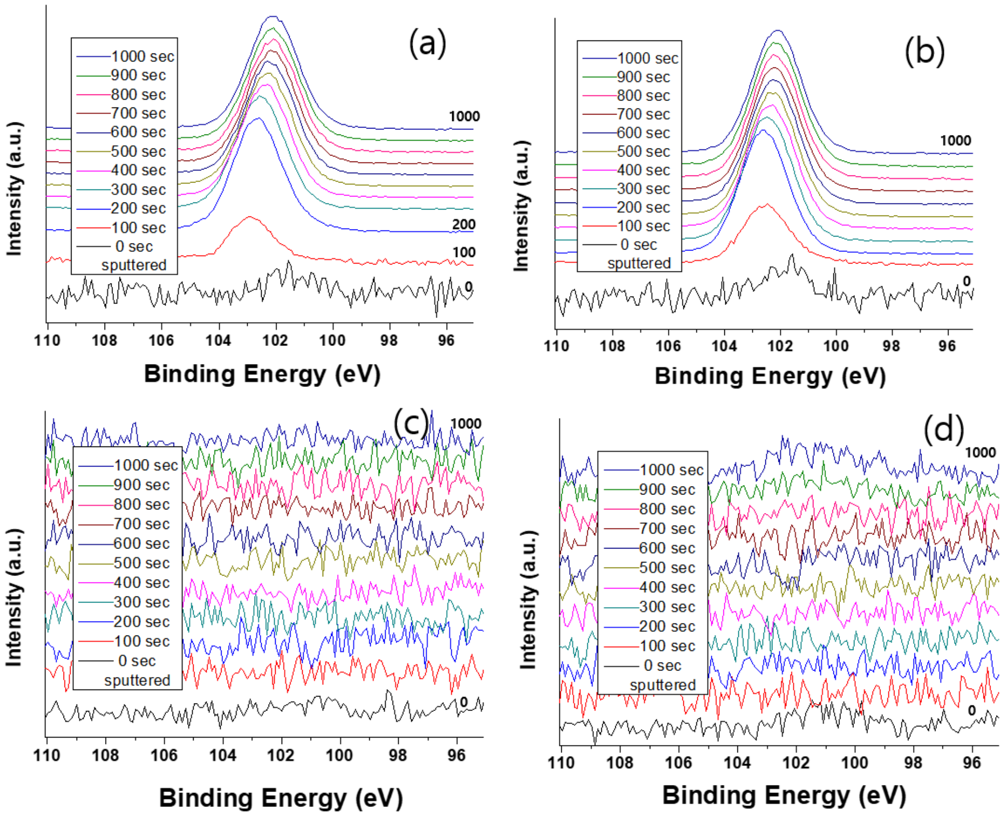

Figure S2. Si2p XPS spectra of (a) PEDOT:PSS, (b) PEDOT:PSS-HT, (c) TA-dipped PEDOT:PSS, (d) TA-dipped PEDOT:PS HT films. The legends indicate the time since start of depth profiling. 0 is equivalent to a standard surface scan.

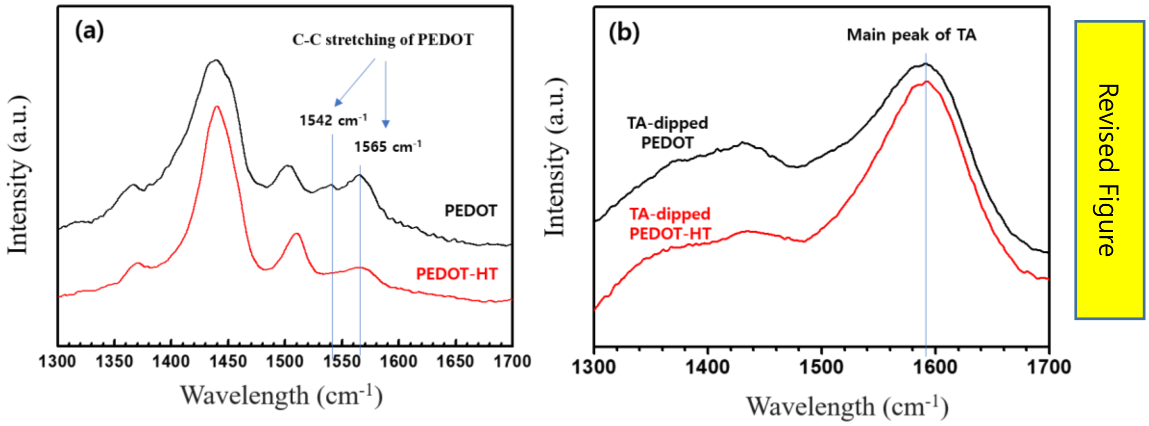

Figure S3. RAMAN spectrum of (a) PEDOT:PSS (black), and heated PEDOT:PSS (red) and (b) TA-dipped PEDOT:PSS (black) and heated TA-dipped PEDOT:PSS (red).
